# Social Anxiety Symptoms in Young Children: Investigating the Interplay of Theory of Mind and Expressions of Shyness

**DOI:** 10.1007/s10802-016-0206-0

**Published:** 2016-09-24

**Authors:** Cristina Colonnesi, Milica Nikolić, Wieke de Vente, Susan M. Bögels

**Affiliations:** 1 University of Amsterdam, Nieuwe Achtergracht 127, 1001 NG Amsterdam, The Netherlands; 2 Research Priority Area Yield, University of Amsterdam, Amsterdam, The Netherlands

**Keywords:** Social anxiety, Childhood, Theory of mind, Expressions of shyness, Shyness

## Abstract

Children’s early onset of social anxiety may be associated with their social understanding, and their ability to express emotions adaptively. We examined whether social anxiety in 48-month-old children (*N* = 110; 54 boys) was related to: *a)* a lower level of theory of mind (ToM); *b)* a lower proclivity to express shyness in a positive way (adaptive); and *c)* a higher tendency to express shyness in a negative way (non-adaptive). In addition, we investigated to what extent children’s level of social anxiety was predicted by the interaction between ToM and expressions of shyness. Children’s positive and negative expressions of shyness were observed during a performance task. ToM was measured with a validated battery, and social anxiety was assessed using both parents’ reports on questionnaires. Socially anxious children had a lower level of ToM, and displayed more negative and less positive shy expressions. However, children with a lower level of ToM who expressed more positive shyness were less socially anxious. Additional results show that children who displayed shyness only in a negative manner were more socially anxious than children who expressed shyness only in a positive way and children who did not display any shyness. Moreover, children who displayed both positive and negative expressions of shyness were more socially anxious than children who displayed shyness only in a positive way. These findings highlight the importance of ToM development and socio-emotional strategies, and their interaction, on the early development of social anxiety.

Children can develop social anxiety symptoms already at an early age, with possible important negative consequences for their social and emotional functioning (Beesdo et al. 2009; Edwards et al. 2010; Stein et al. 2001). Social anxiety refers to the fear or worry of being negatively evaluated during social interactions or social performance situations. If the anxiety is persistent and excessive, and substantially interferes with day-to-day life, it meets the criteria for a social anxiety disorder (DSM-5; American Psychiatric Association 2013). Social anxiety disorder typically starts in childhood, and the lifetime prevalence is estimated to be approximately 8–13 % (Iverach and Rapee 2014). Individual early socio-cognitive development, and specifically theory of mind (ToM), may play an important role in the development of social anxiety. ToM is the capacity to understand and to predict behaviors on the basis of mental states such as desires, intentions, emotions, beliefs, and false-beliefs (Wellman 1990; Wellman and Liu 2004). This ability does not only make social interactions possible, but also helps people to be more successful and popular in social life (Denham 1986). A deficit in ToM in early childhood is related to negative outcomes such as internalizing symptoms, and can enhance the risk of developing social anxiety disorder (Banerjee and Henderson 2001). Presumably, socio-emotional development, such as children’s capacity to have socially adequate reactions and to regulate their emotions during social situations, also plays an important role in social anxiety. Recent findings showed that young children’s proclivity to express shyness in a positive way may help them regulate their social anxiety (Colonnesi et al. 2014). In the present study, we investigated to what extent children’s ToM, and their tendency to regulate their social fear by expressing their shyness in a positive (adaptive) or negative (non-adaptive) way during social situations relate to social anxiety symptoms.

## ToM and the Development of Social Anxiety

From a constructivist approach, children’s capacity to understand and to treat others as independent mental agents begins in early infancy, thanks to early social interactions with the parents and other significant caregivers (Carpendale and Lewis 2004). Implicit ToM abilities in infancy, such as intentional communication (e.g., pointing gesture), non-verbal understanding of intentions and desires, are found to predict later ToM in childhood (Brooks and Meltzoff 2015; Colonnesi et al. 2008; Wellman et al. 2008). By the age of four, due to language acquisition, children attain an explicit ToM, becoming able to predict and to explain others’ behaviors in terms of inner states using and understanding language (Wellman and Liu 2004). At this age, children possess basic ToM abilities such as being able to pretend, the understanding of basic emotions, and the understanding of the difference between reality and non-reality. At the same time, more advanced ToM abilities are still developing, which encompass the understanding of others’ beliefs and false beliefs (Muris et al. 1999; Wellman and Liu 2004). These abilities have a key role in the socio-emotional development from early childhood to adolescence (Carpendale and Lewis 2004).

A normal ToM development seems to be necessary for a healthy socio-emotional development. Several studies illustrated that a good ToM during childhood is related to social competences (Hughes et al. 2006; Zerwas et al. 2004), social perspective taking (Harwood and Farrar 2006), prosocial behavior (Caputi et al. 2012), and school success (Lecce et al. 2011; Trentacosta and Izard 2007). Conversely, deficits in ToM are documented to be associated with autism spectrum disorder (Baron-Cohen 1989), externalizing disorders in childhood (Olson et al. 2011), schizophrenia (Biedermann et al. 2012), and borderline traits in adolescence (Sharp et al. 2011). These findings suggest that individuals with ToM deficits have a lesser understanding of what they can expect from other people, and they are less able to cope in an adaptive way with complex social situations.

Can a deficit in ToM be associated to social anxiety? ToM development chronologically precedes the onset of social anxiety disorder. Hence, a low level of ToM in early childhood could lead to less adaptive manners of participating in social situations, more negative social experiences (e.g., neglect, rejection), less social self-confidence, and possibly a greater level of avoidance and social anxiety (Carpendale and Lewis 2004). A bidirectional influence, however, should also be considered. Social avoidance could prevent or limit social experiences and therefore hamper the development of social understanding (Asendorpf 1990a; Rubin et al. 1990; Suway et al. 2012). In addition, anxious persons may be so hyper-focused on fearing, controlling, and avoiding their own anxious thoughts and feelings that they pay less attention and consequently understand less of others’ mental states (Clark and Wells 1995; Kashdan and Weeks 2010).

Several studies point to an association between ToM and social anxiety. The meta-analysis of O’Toole et al. (2013) shows that children and adolescents with a high level of social anxiety or with a social anxiety disorder are less able to recognize emotions, which is an essential aspect of ToM. Similarly, 6-to-11-year-old children with greater levels of social anxiety and shy negative affect (i.e., self-blaming tendency and low self-esteem) have been found to present deficits in the understanding of emotions, intentions, and beliefs in social situations (Banerjee and Henderson 2001). Alike, Muris and Broeren (2009) found that a low level of ToM (measured with a ToM battery) was associated with more inhibited behaviors during performance situations and interactions with peers and adults in 4-to-9-year-old children (as reported by parents). Other studies, using similar procedures, failed to find a relation between ToM and social anxiety. Colonnesi et al. (2010) found in 4- to 9-year-old children no relation between false-belief understanding and their level of social anxiety. Similarly, Broeren et al. (2013) reported that ToM (measured with a ToM battery) did not predict a social anxiety trajectory in children from 4 to 9 years of age. In sum, the above mentioned studies offer inconsistent findings on the relation between ToM and social anxiety. It seems, however, that a low understanding of emotions, rather than beliefs, might be associated to social anxiety symptoms.

## Expression of Shyness and the Relation to Social Anxiety

The relation between ToM and social anxiety may be influenced by children’s ability to regulate their shyness (Asendorpf 1990b; Colonnesi et al. 2014; Henderson and Zimbardo 2001; Lewis 2001). Shyness occurs in social situations in which individuals are confronted with social attention or evaluations, and can be qualified as a state (situational shyness) or as a trait (Asendorpf 1989; Buss 1986; Colonnesi et al. 2014; Eggum-Wilkens et al. 2015; Henderson and Zimbardo 2001; Lewis 2001; Reddy 2005; Rubin et al. 2009). State shyness is the emotional and cognitive experience of shyness in response to a specific threatening social situation. Everyone can experience shyness to some extent, and we can find individual differences in the gradation and in the modality to express shyness (Asendorpf 1990b). Trait shyness, conversely, refers to the recurrent and persistent experience of shyness, and is normally qualified as a temperamental or personality dimension (Buss 1980). The level of state shyness of people with high trait shyness is supposed to be higher than the level of state shyness of people low in trait shyness.

Shyness is typically manifested by specific shy facial expressions, disorganized behavior, or physiological reactions such as blushing (Asendorpf 1990b; Buss 1986; Henderson and Zimbardo 2001; Lewis et al. 1991). When experiencing shyness people are often concerned or worried about being socially exposed to others’ evaluations but, at the same time, they wish to remain engaged in the situation and to make a good impression (Asendorpf 1990a; Buss 1986; Leary et al. 1995; Schlenker and Leary 1982). The essence of shyness is therefore an approach-avoidance conflict during social situations (Asendorpf 1990a, 1990b). Recent observational studies distinguished between positive and negative facial expressions of shyness (Colonnesi et al. 2014; Nikolić et al. 2016). While positive expressions of shyness could be seen as the manifestations of approach-ambivalent shyness, negative expressions of shyness are more an avoidant-ambivalent type of shyness. Both expressions of shyness seem to be involuntary behavioral reactions since they happen suddenly during social interactions, and the facial expression is about 2–3 s long. They appear as abrupt reactions to reduce arousal.

Children’s positive shyness is expressed by positive facial expressions (i.e., smiles) in combination with gaze or head aversions (Asendorpf 1990b). These expressions are also defined as “coy smiles” and are produced in flirting situations as well (Hall and Xing 2014; Moore 2010). Coy smiles can already be observed during early infancy (Colonnesi et al. 2013; Reddy 2000), in particular when infants are exposed to the attention of novel persons. At 2.5 years, the same expressions have been found to be associated with sociability, and with a lower level of social anxiety in children (Colonnesi et al. 2014). In children aged four-and-a-half, positive shy expressions were related to less social anxiety and were found to serve a protective role in the association between blushing and social anxiety (Nikolić et al. 2016). To conclude, positive expressions of shyness are behavioral manifestations of children’s capacity to regulate their ambivalent feelings and fear during social situations. In addition, to express shyness in a positive manner seems to serve as an appeasement function in social interactions.

In contrast, negative expressions of shyness are combinations of gaze and head aversions during negative facial expressions (i.e., a frown). These facial expressions are included in the criteria of social behavioral inhibition (BI) which refers to fear or wariness regarding novel people or social situations (Buss and Goldsmith 2000; Goldsmith et al. 1993). BI is normally expressed by avoidant behavior or hesitancy, gaze and head aversion, and vocal distress occurring during negative facial expression of sadness or fear (Buss and Goldsmith 2000). BI has been found to be a risk factor for internalizing difficulties, and in particular for social anxiety (Biederman et al. 2014; Buss et al. 2013; Clauss and Blackford 2012; Hirshfeld-Becker et al. 2007; Volbrecht and Goldsmith 2010). Colonnesi et al. (2014) found that toddlers’ negative facial expressions, with and without gaze and head aversions, were related to a lower level of sociability. Possibly, children express shyness in a negative way when they are not able to regulate their ambivalent emotions and fear in an adaptive way in social situations. In these cases, avoidance becomes dominant in the approach-avoidance conflict. According to Colonnesi and colleagues the negative expressions of shyness are possibly associated to social inhibition, early experience of social failure, interpersonal rejection, and social anxiety (disorder).

Although shyness and social anxiety are found to be related, to be shy does not imply per se to be socially anxious (Rapee 2010). First, while social anxiety disorder is a clearly defined syndrome (DSM-5; American Psychiatric Association 2013), different definitions have been provided about shyness, both as a state emotion and as a personality trait (Reddy 2005). Second, an extensive body of research shows that the percentage of people considering themselves shy is consistently higher than the percentage of people meeting the criteria for social anxiety disorder (Burstein et al. 2011; Chavira et al. 2002; Costello et al. 2003; Ford et al. 2003; Heiser et al. 2003; Rapee et al. 2009). Moreover, shyness severity has been found only to account for 22 % of the variance in social anxiety disorder (Heiser et al. 2003). There are two main perspectives about the relation between shyness and social anxiety disorder. According to the first perspective they are part of continuum where social anxiety disorder is the result of an extreme or clinical form of shyness (Chavira et al. 2002; Marshall and Lipsett 1994; McNeil 2001). This perspective supports the notion that shyness is a normal facet of personality and that it is not necessarily pathological (Carducci 1999). According to the second perspective shyness and social anxiety disorder are two partly overlapping constructs with shyness being a broader and more heterogeneous construct than social anxiety disorder (Heiser et al. 2003). In this case, shyness and social anxiety not only vary in degree but are also qualitatively different. The possibility to express shyness, but not anxiety, in a positive or in a negative way confirms the idea that shyness is a broader construct than social anxiety.

A developmental interplay can be expected between the experience and expression of shyness and ToM in childhood. According to a Piagetian perspective, the experience of shyness seems to be a determinant part of the self-consciousness development because it requires the ability to reflect on the self as seen by others, and to be capable of concern about social evaluation (Arkin et al. 1986; Asendorpf 1986; Lewis 1995; Selman and Byrne 1974). Moreover, both the development of self-consciousness and the onset of an explicit ToM start by the age of 4–5 years (Buss 1986; Yuill and Banerjee 2001). We would therefore predict that, by the age of 4.5 years, children’s level of ToM as well as their proclivity to display shyness in a positive or in a negative manner, and their interaction, are possible indicators of children’s early level of social anxiety.

## The Present Study

The aims of the present study were twofold. First, we investigated how, in children of 4.5 years old, ToM and shyness expressed in positive and negative ways were associated with the level of social anxiety as reported by parents. We expected a deficit in ToM, as well as a high level of negative expressions of shyness, to be related to a greater level of social anxiety, and we expected positive expressions of shyness to be associated with lower social anxiety. Second, we explored the interplay between children’s ToM and children’s positive and negative expressions of shyness on their level of social anxiety. While children’s tendency to express shyness in a positive way was expected to reduce the association between deficits in ToM and social anxiety levels, children’s tendency to express shyness in a negative way was expected to enhance the association between ToM deficits and social anxiety levels.

In order to avoid shared-method variance, test data were combined with observational data and parental reports. Children’s level of ToM was assessed with a validated ToM battery, their positive and negative expressions of shyness were observed during a singing performance, and children’s facial expressions were systematically coded using the coding system of Colonnesi et al. (2014). Children’s level of social anxiety was assessed with both parents’ reports on questionnaires.

## Method

### Participants

The original sample consisted of 151 firstborn children and their families who were part of an ongoing longitudinal study on the development of social anxiety in children at the University of Amsterdam. When the child was 4.5 years old, 118 children and their parents participated in the present study. As eight children did not participate in the lab measurements, the final sample consisted of 110 children (54 boys) who had an average age of 53.46 months (*SD* = 1.70). Families were recruited during the pregnancy of their first child through midwives, advertisements and leaflets. Parents were mostly Caucasian (93 %) from middle-high socio-economic status and with a relatively high educational level, *M* = 6.84, *SD* = 1.16 on a scale of 1 (*primary school*) to 8 (*university*). Participants were all healthy, full-term children with no pre- or post-natal medical histories. The study was reviewed by the Research Ethical Committee of the University of Amsterdam. In order to participate in the study written consent of both the parents was required.

### Measures and Procedure

Both parents visited the lab separately when their child was 4.5 years old. Children’s ToM was assessed with a shortened version of the TOM-test-r (Muris et al. 1999; Steerneman et al. 2009) during the measurement with the mother. The performance and self-watching tasks were conducted during the lab visit with the father. Two weeks before the lab measurement both parents completed the Dutch version of the revised Preschool Anxiety Scale (PAS-R; Edwards et al. 2010) as a measure of children’s level of social anxiety. Children received a small present in return for their participation, and parents received a 20 euro gift voucher, and a DVD of the laboratory session.

### Theory of Mind (ToM)

The TOM-test-r interview (Muris et al. 1999; Steerneman et al. 2009) evaluates ToM abilities from three to 12 years of age. The test includes 14 short illustrated stories about which the child has to answer 36 questions. The test consists of three subscales: (1) ToM1, tapping into a basic level of ToM with: pretense (e.g., “Do as if you brush your teeth”), the difference between reality and non-reality (e.g. “Can people see a bicycle you are dreaming about?”), and recognition of basic emotions (e.g., “Who in this picture is angry?”); (2) ToM2, about understanding of beliefs: the first order belief (e.g., What children think about real events, “Peter thinks that Sue is sad”), and the first order false-belief (e.g., the “Smarties test”); and ToM3, about more advanced aspects of ToM (e.g., second-order belief, understanding of humor). ToM3 was not assessed in the present study, because children were not expected to master an advanced level of ToM yet. The ToM-test is a reliable and valid measure demonstrating sufficient to good internal consistency, test-retest stability, and inter-rater reliability (Muris et al. 1999). The interviews with the children were digitally video-recorded and coded by three master student observers after an extensive training (κ > 0.80). Internal consistency in the present study was close to acceptable, α = 0.67 for ToM1, and α = 0.67 for ToM2, probably owing to the multidimensionality of the instrument and to the low number of the subscales (3 for ToM1 and 2 for ToM2). The average inter-rater reliability, assessed using 22 double-coded observations (20 %) (κ) was: ToM1, *к* = 0.99, ToM2, *к* = 0.92.

### Performance and Self-Watching Tasks

The performance task and the self-watching task were conducted in order to elicit children’s positive and negative expressions of shyness. During the performance task children were asked to sing a song in front of a small audience: the experimenter (E1), their father, and a second novel experimenter (E2) who recorded the performance with a high definition video-camera. First, children were invited to choose a costume and to stand on a podium with a spotlight and a microphone. Next children were told that someone was coming to the room to record their performance in order to make a video as a gift for the mother (E2). Children were then invited to sing a song they liked. The experimenter introduced the child saying: “And now, the famous pop-star [name of the child] will sing for us [name of the song]!” After the performance the audience applauded and the child was complimented. During the self-watching task children were asked to sit on the podium and watch their recorded performance on a television screen with their father, E1, and E2. The video of the performance situation recorded by the remote camera was played until the applause. The mean duration of the performance task and of the self-watching task were 77.89 s (*SD* = 35.33) and 56.60 s (*SD* = 34.62), respectively.

Of the 110 children who visited the lab with the father, nine refused to participate in the performance task. Therefore, observational measures for these children were not available. Of the 101 children who participated in the performance task, 81 children sang a song on stage, and 20 did not sing. Only the children who sang on stage watched their performance because of ethical reasons.

### Coding the Performance and Self-Watching Tasks

The validated coding system of Colonnesi et al. (2014) was used to code children’s expressions of positive and negative shyness. Differently from observational methods to assess BI (Goldsmith et al. 1993), this coding system focus only on the coding of facial expressions, and it comprises two dimensions of shyness: an approach-ambivalent shyness (positive shyness), and an avoidant-ambivalent type of shyness (negative shyness). The coding of the performance task started after E1 introduced the child (also when the child did not sing) and lasted for 60 s (for children whose performance lasted for less than 60 s, a corrected number of behaviors was calculated). The coding of the self-watching task started as soon as the video started and the child began watching the video. The observation of the self-watching task ended after 60 s. The Observer XT 11.5 event-logging software (Noldus et al. 2000) was used to code the video observations. Children’s positive, neutral, and negative facial expressions were coded as state events (i.e., behaviors that take a period of time). Apex, gaze and head aversions were coded as point events (i.e., a behavior that only takes an instant in time). The observations were coded by five independent master student observers and one doctorate student after extensive training (*к* > 0.80). Three observers coded the performance task, and three observers coded the self-watching task.

**Fig. 1:**
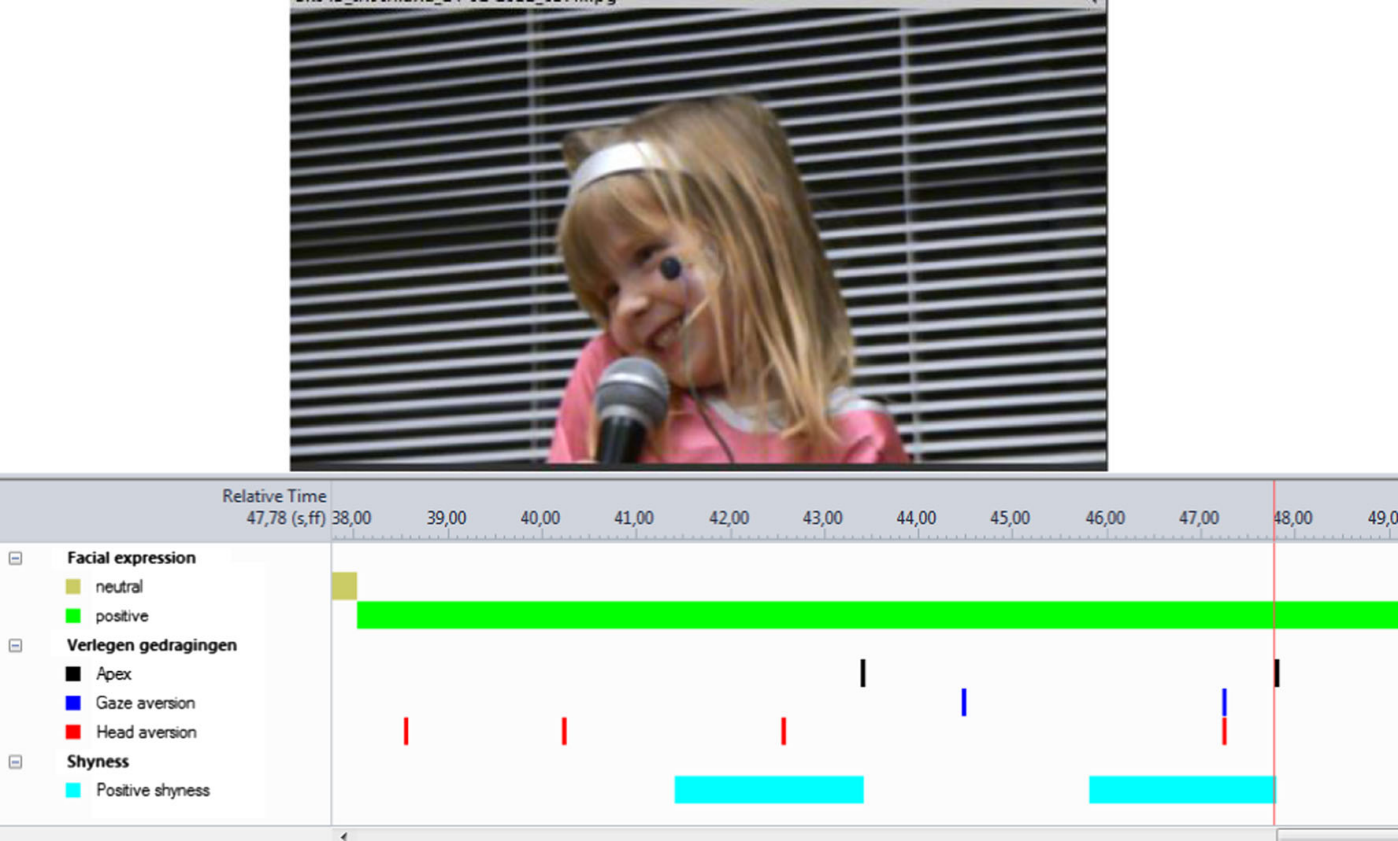
Example of child displaying positive expressions of shyness and the visualization of the data through the observer

Using the analysis function of The Observer, two target behaviors were obtained combining the state and the point events through nesting and lag-sequential analyses: positive expressions of shyness (number of positive facial expressions in which an aversion of gaze, head, or both occurred within 2–0.0 s prior to the apex of the smile); and negative expressions of shyness (number of negative facial expressions in which an aversion of gaze, head, or both occurred in a temporal episode of 2 s). Figure 1 shows a visualization of two expressions of positive shyness. The first expression occurs by the presence of a head aversion (42.5 s) 0.8 s before the apex (43.4 s). The second positive expression of shyness occurs by a co-occurrence of head and gaze aversion (47.2 s) 0.6 s before the apex (47.8 s). The second expression is shown in the picture above the visualization. The Inter-rater reliability was calculated for 18 observations (20 %) of the performance task, and for 18 observations (27 %) of the self-watching task. Cohen’s kappa corrected for kappa max (Bakeman et al. 2005) was *к* = 0.89 for the performance task, and *к* = 0.95 for the self-watching task.

When exporting the data from Observer, enough individual variance was found for children’s expression of shyness in the performance task but not in the watching-back task. During the self-watching task, 29 children showed positive expressions of shyness one time and one child did so three times, and only two children displayed a negative expression of shyness once. Because of the low frequency of children’s expressions of positive and negative shyness in the self-watching task, only children’s expressions of shyness during the performance task were used for the analyses.

### Level of Social Anxiety

Social anxiety was measured with the social anxiety subscale of the Dutch version of the revised Preschool Anxiety Scale (PAS-R; Edwards et al. 2010). The subscale consists of seven items (e.g., “Acts shy and quiet around new people”) rated from 0 (*not at all true*) to 4 (*very often true*). The subscale has good construct validity and internal consistency (Edwards et al. 2010). Intra-scale homogeneity for social anxiety in this study was α = 0.87 for mother and α = 0.88 for father, and the correlation between parents was *r*(96) = 0.49, *p* < 0.001. A composite standardized score of mothers’ and fathers’ reports of children’s social anxiety was computed and used in the analyses.

### Data Inspection and Analytic Strategy

Nine children (8.20 %) explicitly refused to sing, therefore, no performance data were available for these children. These children did not differ from the children who did perform in their level of ToM1 levels, *t*(105) = −0.53, *p* = 0.596, *d* = −0.18 (*M*_not performing_ = 8.00; *SD* = 1.12; *M*_performing_ = 8.31; *SD* = 1.69), ToM2 level, *t*(98) = 0.64, *p* = 0.949, *d* = 0.02 (*M*_not performing_ = 4.25; *SD* = 2.44; *M*_performing_ = 4.20; *SD* = 2.28), and social anxiety, *t*(100) = 0.78, *p* = 0.437, *d* = 0.26 (*M*_not performing_ = 2.26; *SD* = 0.57; *M*_performing_ = 2.08; *SD* = 0.69). A total sample of 101 children was used for the analyses.

Five observations (5 %) of the performance task were missing because of procedural errors or problems with video recording. Due to procedural errors 3 % of ToM1 and 9 % of ToM2 data were missing, and 8 (7 %) scores of the PAS-R scale were missing because both parents did not fill in the questionnaire. Analysis of missing data showed a total of 5.94 % missing values, and these were distributed randomly in the database: the Little MCAR test was not significant, χ^2^(18) = 16.73, *p* = 0.542. Missing values were handled using the SPSS 22 estimation maximization (EM) procedure (Graham 2009). All the analyses were conducted twice: the original data and the data with imputed data provided similar results. Results with the imputed data are presented in the Result section.

Next, data were assessed for skewness and kurtosis. Social anxiety and ToM scores (ToM1 and ToM2) were normally distributed, but children’s number of expressions of shyness (negative and positive) were not. A log transformation was applied (Field 2005) on these two variables to improve the distributions, skewness_positive shyness_ = 0.92 (*SE* = 0.24); skewness_negative shyness_ = 1.59 (*SE* = 0.24).

In order to examine the relations between ToM, expressions of shyness, and level of social anxiety, Pearson’s correlation analyses were conducted. To assess the extent to which children’s levels of ToM, the way they express shyness, and the interaction between ToM and expressions of shyness affected their level of social anxiety, multiple moderator analyses were conducted on children’s level of social anxiety with children’s ToM1 (first regression) and ToM2 (second regression) as focal predictors, and children’s positive and negative expressions of shyness as moderators of ToM effect. Preliminary VIF statistics indicated no multicollinearity (VIF = 1.30 for ToM1, 1.31 for ToM2, 1.21 f or positive expressions of shyness, and 1.33 for negative expressions of shyness). Analyses were performed using SPSS statistic software and the macro PROCESS (Hayes 2013). Moderation model = 2 was used (5000 bootstrap samples), and the scores of ToM and expressions of shyness were standardized prior to the analyses. Moderation (i.e., an interaction) occurs when the size or direction of a predictor variable’s effect on an outcome variable depends on the value of the moderator variable. Significant interactions were probed using the Pick-a-point techniques via the PROCESS script for SPSS. The Pick-a-point technique allowed us to ascertain whether ToM was related to social anxiety among children who produced a low number of expression of shyness (1 *SD* below the mean), medium number (mean), and high number (1 *SD* above the mean). Children’s levels of ToM and social anxiety were additionally explored using a MANOVA with groups of children based on their expression of shyness as a between factor.

## Results

### Preliminary Results

A preliminary MANOVA was conducted in order to explore the effect of children’s gender on children’s level of ToM1, ToM2, positive and negative expressions of shyness, and social anxiety. The multivariate test did not reach significance, *F*(95, 5000) = 1.47*, p* = 0.207, η_p_^2^ = 0.072, as well as the univariate analyses, range *F*(1, 98) = 2.97 to 0.00; range *p* = 0.088 to 0.983, range η_p_^2^ = 0.029 to < 0.000. Children’s gender was therefore not included in the analyses.

### Relation between ToM, Expressions of Shyness, and Social Anxiety Level

Descriptive statistics and Pearson’s correlations between children’s ToM, their positive and negative expressions of shyness, and their level of social anxiety are reported in Table 1. Children’s ToM1 was negatively associated to the negative expressions of shyness, and to social anxiety. ToM2, but not ToM1, was positively associated to positive expressions of shyness. In addition, a positive relation was found between negative expressions of shyness and social anxiety. Positive expressions of shyness were negatively correlated to negative expressions of shyness and to social anxiety.

**Table 1:** Descriptive statistics and correlations (p values) of ToM1,ToM2, positive and negative expressions of shyness, and levels of social anxiety (N = 101)

	*M (SD)*	*Range*	2.	3.	4.	5.
1. ToM1	8.31 (1.67)	3–11	0.35	0.09	-0.27	-0.32
			(<0.001)	(0.365)	(0.007)	(0.001)
2. ToM2	4.16 (2.19)	0–10	-	0.20	0.12	-0.06
				(0.046)	(0.226)	(0.562)
3. Positive Expressions of Shyness	2.23 (2.35)	0–11		-	-0.33	-0.25
					(0.001)	(0.012)
4. Negative Expressions of Shyness	2.07 (3.82)	0–19			-	0.32
						(< 0.001)
5. Social anxiety	2.07 (0.66)	1–4				-

ToM1: Basic level of theory of mind; ToM2: Understanding of beliefs

### Level of ToM1 and ToM 2 and Expressions of Shyness as Predictors of Social Anxiety

A first linear regression model was conducted to test the predictive role of the interaction between ToM1 and positive and negative expressions of shyness on children’s social anxiety. Table 2 reports the partial standardized coefficients for the main variables, the interactions terms, and the R-square increase due to interaction. The regression model was found to be significant. ToM1, positive expressions of shyness, and their interaction significantly predicted children’s social anxiety. Children’s negative expressions of shyness, conversely, and the interaction between negative expressions of shyness and ToM1 did not predict social anxiety.

**Table 2:** Multiple regression analyses with social anxiety as dependent variable, ToM1 and ToM2 as predictors, and positive and negative expressions of shyness as moderators (N = 101)

	*b (SE)*	*t*	*p*	IC 95 %	R2	*F*	*p*	ΔF2	*F*	*p*
*First regression ToM1*					0.22	5.49	<0.001			
ToM1	-0.10 (0.04)	-2.63	0.010	-0.17, −0.02						
Positive Shyness	-1.67 (0.84)	-1.99	0.049	-3.34, −0.01						
Negative Shyness	0.88 (0.61)	1.45	0.150	-0.32, 2.09						
ToM1 x Positive Shyness	1.08 (0.52)	2.08	0.041	0.05, 2.11				0.04	4.32	0.041
ToM1 x Negative Shyness	0.01 (0.35)	0.02	0.988	-0.69, 0.69				>0.00	0.00	0.988
Both interactions								0.04	2.48	0.089
*Second regression ToM2*					0.13	2.85	0.019			
ToM2	-0.02 (0.03)	-0.75	0.458	-0.08, −0.04						
Positive Shyness	-1.30 (0.90)	-1.43	0.155	-3.09, 0.50						
Negative Shyness	1.66 (0.63)	2.64	0.010	0.41, 2.91						
ToM2 x Positive Shyness	0.27 (0.42)	0.65	0.518	-0.57, 1.11				<0.01	0.42	0.518
ToM2 x Negative Shyness	0.07 (0.31)	0.22	0.823	-0.55, 0.69				<0.01	0.05	0.823
Both interactions								<0.01	0.21	0.809

ToM1: Basic level of theory of mind; ToM2: Understanding of beliefs

Probing the ToM1 as predictor and positive expression of shyness as moderator interaction with the pick-a-point approach revealed that level of ToM1 was significantly and negatively related to level of social anxiety among children who showed low (no positive expressions of shyness; *n* = 29), *b* = −0.20, *SE* = 0.05, *t*(97) = −4.19, *p* < 0.001, 95 % CI [−0.30, −0.11], and medium numbers of positive expressions of shyness (1–4 positive expressions of shyness; *n* = 52), *b* = −0.11, *SE* = 0.04, *t*(97) = −3.08, *p* = 0.003, 95 % CI [−0.18, −0.04], but not among children who showed high numbers of positive expressions of shyness (5 or more positive expressions of shyness; *n* = 20), *b* = −0.02, *SE* = 0.05, *t*(97) = −0.34, *p* = 0.738, 95 % CI [−0.12, 0.09]. The three groups of children differed significantly in the number of positive expressions of shyness, *F*(2, 100) = 365.20, *p* < 0.001 (Bonferroni’s post-hoc < 0.001).

**Fig. 2:**
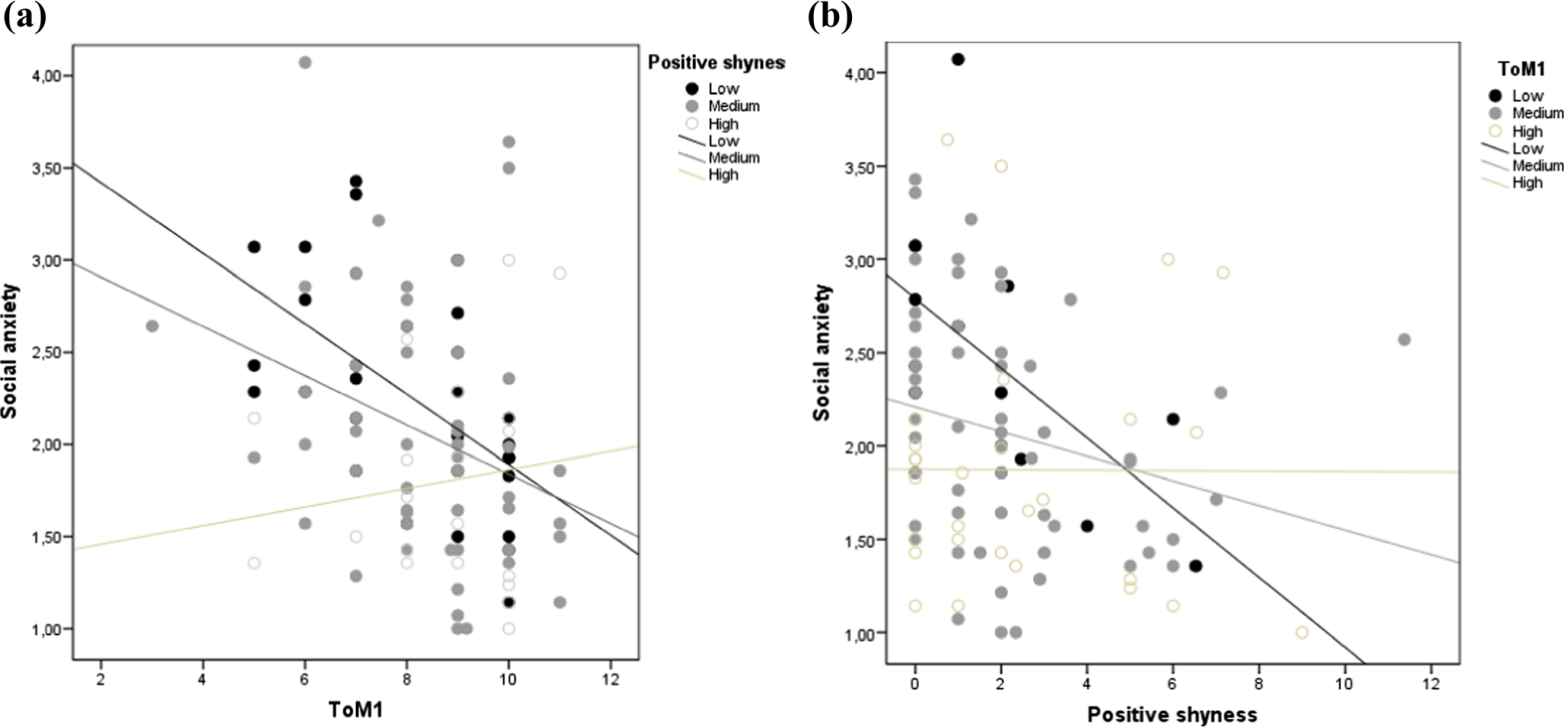
**a** Simple slopes of ToM predicting level of social anxiety for 1 *SD* below the mean (Low), the mean (Medium), and 1 *SD* above the mean (High) of positive expressions of shyness; **b**. Simple slopes of positive expressions of shyness predicting level of social anxiety for 1 *SD* below the mean (Low), the mean (Medium), and 1 *SD* above the mean (High) of basic level of ToM1. Note. ToM1: basic level of theory of mind

Probing the same interaction with the number of positive expressions of shyness as predictor and ToM1 as moderator yielded similar results. The expressions of positive shyness was significantly and negatively related to social anxiety among children who showed low (ToM1 score between 0 and 6; *n* = 15), *b* = −4.11, *SE* = 1.16, *t*(97) = −3.54, *p* < 0.001, 95 % CI [−6.41, −1.80], and medium level of ToM1 (ToM1 score between 7 and 9; *n* = 58), *b* = −2.08, *SE* = 0.78, *t*(97) = −2.66, *p* = 0.009, 95 % CI [−3.64, −0.53], but not among children who showed high level of ToM1 (ToM1 score higher than 9; *n* = 28), *b* = −0.06, SE = 1.06, *t*(97) = −0.05, *p* = 0.958, 95 % CI [−2.17, 2.05]. Figure 2 illustrates both interaction effects.

A second regression model was conducted to test the moderation effect of positive and negative expressions of shyness on the relation between ToM2 and social anxiety (Table 2). The regression model was significant. Children’s use of negative expressions of shyness was the only significant predictor of children’s social anxiety in this model. Both moderation effects (positive and negative expressions of shyness) did not reach significance.

### Groups on the Expressions of Shyness

In order to further explore the data using a person-oriented approach, four groups were formed on the basis of the expressions of shyness. Fourteen children never showed positive or negative shyness facial expressions (no-shy children), 48 children showed no negative shy expressions and one or more positive shy expressions (positive-shy children), 24 children displayed both positive and negative shyness more than one time (mixed-shy children), and 15 children showed no positive shy expressions and one or more negative shy expressions (negative-shy children). The MANOVA revealed a significant difference between these four groups in their level of ToM1 and social anxiety, *F*(95, 291) = 3.13, *p* = 0.001, η_p_^2^ = 0.089. Descriptive statistics for the four groups and the test of between-subjects effects are reported in Table 3. Although a significant effects was found for ToM1, post-test (Sidak) comparisons showed no significant differences between groups. A second significant effect was found for children’s level of social anxiety. The post-test comparisons revealed that negative-shy children had a significantly higher social anxiety than positive-shy children and non-shy children. Moreover, social anxiety was higher in mixed-shy children than in positive-shy children. In conclusion, children who expressed their shyness in a negative way, also when combined with positive expressions of shyness, had greater levels of social anxiety than children who expressed their shyness in a positive manner or children who never expressed shyness.

**Table 3:** Results of MANOVA performed for the four groups (Number of subjects for the analysis) on the expressions of shyness on the measure of ToM1, ToM2, and social anxiety. significance levels of Sidak comparisons are reported in the note

	ToM1		ToM2		Social Anxiety	
	*M*	*(SD)*	*M*	*(SD)*	*M*	*(SD)*
No-shy (*n* = 14)	8.71	(1.64)	3.24	(1.77)	1.93^a^	(0.43)
Positive shy (*n* = 48)	8.69	(1.52)	4.28	(2.45)	1.84^b, c^	(0.61)
Mixed-shy (*n* = 24)	7.85	(1.78)	4.52	(1.84)	2.29^b^	(0.71)
Negative shy (*n* = 15)	7.47	(1.64)	4.09	(2.11)	2.57 ^a, c^	(0.55)
*F*	3.17		1.11		6.86	
*p*	0.028		0.351		< 0.001	
η2	0.089		0.033		0.175	

^a^ *p* = 0.036, 95 % CI [−1.25, −0.03]

^b^ *p* = 0.024, 95 % CI [−0.86, −0.04]

^c^ *p* = 0.001, 95 % CI [−1.21, −0.24]

## Discussion

The present study was unique in examining both ToM and expressions of shyness as indicators of children’s level of social anxiety in early childhood. First, we found that early social anxiety symptoms are associated with a low basic level of ToM (ToM1), a low use of positive expressions of shyness, and a high use of negative expressions of shyness during socially stressful situation such as public performance. Second, we explored to what extent the interplay between children’s ToM and the use of negative and positive expressions of shyness was associated with social anxiety. We found an interplay between children’s ToM and positive expressions of shyness in relation to social anxiety. More specifically, children’s social anxiety was not related to a low level of ToM in children with a high proclivity to express shyness in a positive way. Similarly, children’s social anxiety was not associated with children’s level of positive shyness when their level of ToM was high. No interplay was found between children’s ToM and negative expressions of shyness. Third, we compared groups of children on the basis of their production of positive and negative expressions of shyness. Results revealed that children who displayed negative expressions of shyness had greater levels of social anxiety than children who only displayed positive expressions of shyness or no shyness. In addition, similar levels of social anxiety were found in children who displayed shyness only in a negative way and children who displayed a combination of negative and positive expressions of shyness. These results are discussed in terms of their contribution to our knowledge of early development of social anxiety, and with regards to their implication for the future research and the practice.

### ToM and the Relation to Expression of Shyness and to Social Anxiety

Children’s basic level of ToM was found to be negatively related to negative expressions of shyness. These results suggest that a good ToM development in early childhood can facilitate social understanding by promoting positive social experiences, self-confidence, and peer relations. Conversely, a ToM delay or deficit can reduce social understanding, and increase non-adaptive behavior and therefore negative social experiences such as peer rejection (Caputi et al. 2012; Kokkinos et al. 2016; Slaughter et al. 2002). Besides, a more advanced ToM (ToM2) was positively associated to children’s positive expressions of shyness. Possibly, the tendency to express shyness in a positive way is related to a higher level of sociability, which is the tendency to seek and take pleasure in interactions with others (Colonnesi et al. 2014). Sociability stimulates social contacts and social experiences, which therefore should enhance the development of more advanced levels of social understanding.

In line with expectations, children’s low basic ToM was also associated with a high level of social anxiety. This result confirms previous findings on the relation between a deficiency in ToM and social anxiety (Banerjee and Henderson 2001; O’Toole et al. 2013). This result might also offer an explanation for the high levels of social anxiety among children with autism (van Steensel et al. 2011) who present with impairment in appreciating the mental states of other individuals (Baron-Cohen 1989). Note that social-understanding abilities, as well as the expressions of shyness, occur already during infancy (Baillargeon et al. 2010; Reddy 2000), while the earliest onset of social anxiety can be found in early childhood (Edwards et al. 2010). Hence, in the present study we tested the effect of ToM and the expressions of shyness as possible determinants of social anxiety. However, the relation between social understanding and social anxiety may well be bidirectional (Suway et al. 2012). That is, a greater level of social anxiety can be a determinant for less adaptive reactions during social interactions, fewer positive social experiences and therefore, more social avoidance, and less opportunity to further develop an age-appropriate level of ToM (Clark and Wells 1995; Kashdan and Weeks 2010; Rubin et al. 1990).

Unexpectedly and in contrast with the ToM1 association with social anxiety, a lower level of advanced ToM (ToM2), appeared to be unrelated to social anxiety. A possible explanation is that while ToM1 includes more basic abilities for children of 4.5 years, the understanding of belief and false-belief (ToM2) is still developing between the age of 4 and 5 years (Wellman and Liu 2004), and therefore might not yet be relevant for social anxiety development at that age. Another possible explanation is that social anxiety can be better predicted by more general aspects of social understanding such as understanding of emotions and the ability to pretend (ToM1), than by more cognitive-related understanding such as the understanding of beliefs and false-beliefs (ToM2). Hence, fear during social situation can be more related to the inability to understand our own ambivalent feelings and not overestimating others’ expectations than to the incapacity to appreciate others’ thoughts (Kalbe et al. 2010; Tibi-Elhanany and Shamay-Tsoory 2011).

A low level of ToM, and perhaps also advanced ToM, might be expected to be related to social anxiety also at later ages. Empirical evidence shows that, in adolescents and in adults, social anxiety disorder is determined and maintained by different cognitive biases in social-information processing, such as negative beliefs and preoccupation about other people’s evaluations (Clark and Wells 1995; Schlenker and Leary 1982; Voncken et al. 2003), and distorted social interpretations (Miers et al. 2011). Hezel and McNally (2014) found that socially anxious adults performed worse on ToM tests than non-socially anxious adults, attributing more intense emotions and greater meaning to others’ thinking and feeling. On the other hand, at later ages, also a high level of ToM can be a risk factor for social anxiety, as children who are early or advanced at reading others’ mind might be more aware of the possibility of negative evaluation. Tibi-Elhanany and Shamay-Tsoory (2011) found that high socially anxious adults presented a greater level of affective ToM (i.e., making inferences regarding one’s emotional state) than low socially anxious adults. Similarly, a cross-sectional study in children from 3 to 12 years, revealed that, at older age, children with a greater level of ToM were more likely to refuse social performances such as dancing or singing when they can choose a less risky activity instead (Chaplin and Norton 2015). The role of too low and too high ToM in social anxiety, in children as well as adults, is clearly an area for further research.

### Children’s Expressions of Shyness and Social Anxiety

Children’s level of social anxiety was found to be negatively associated with less positive expressions of shyness. This result confirms previous findings of Colonnesi et al. (2014), suggesting that positive, but not negative, expressions of shyness, are an adaptive behavior in social interactions when children are afraid of not being able to meet others’ expectations. In these situations displaying positive shyness, such as producing a coy smile, can be a behavioral predisposition enlisted to manage the experience of emotional arousal (i.e., regulation of shyness), or to alter one’s display of emotion to others (i.e., hiding the discomfort), or both, while to express shyness in a negative way is probably the incapacity of both (Colonnesi et al. 2014). Shy children who express these feelings in a positive way are able to appropriately communicate that they are apprehensive of others’ evaluation, to moderate social contact, and therefore to prevent social negative outcomes, and in particular peer rejection. On a long term perspective, these children can be more socially competent and less socially anxious because they successfully handle social situations.

As expected, children’s social anxiety was related to more negative expressions of shyness. This result is in line with previous findings on the relation between children’s BI and social anxiety (Biederman et al. 2014; Hirshfeld-Becker et al. 2007; Buss et al. 2013; Volbrecht and Goldsmith 2010). This relation can have crucial implication for the socio-emotional development of the children. Hence, children’s early tendency to express shyness in a non-adaptive way during social situations may have possible negative social outcomes at later age. Frequent expressions of negative shyness may cause negative social experiences such as peer rejections and willingness to avoid social contacts with withdrawal as the possible long-term outcome (Coplan and Rubin 2004; Rubin et al. 2009). Social withdrawal refers to the tendency, across situations and over time, to display solitary social behavior, and might be seen as a maladaptive behavior (Coplan and Rubin 2010; Rubin and Asendorpf 1993). Withdrawal has been found to be related to social anxiety in middle childhood and in adolescence (Findlay et al. 2009; Fordham and Stevenson-Hinde 1999; Weeks et al. 2009). Negative expressions of shyness may also be related to social reticence at later age. Lamm et al. (2014), for instance, found that only children with greater level of shyness (i.e., BI score) combined with greater cognitive-control activation at 2–3 years had greater levels of reticence at the age of 7 years.

Post-hoc analyses conducted on four groups of children who displayed (or not) positive and negative expressions of shyness, show no differences between children who only displayed negative shyness and children who displayed both negative and positive shyness (mixed-shy group): both groups had a greater level of social anxiety than children who only displayed positive shyness and non-shy children. These results indicate that both positive and negative shyness can be expressed in the same situation. We should therefore think about positive and negative shyness not as two extremes of a continuum of shyness, but rather as two ways to express a shy emotion which do not automatically exclude each other. The finding that the level of social anxiety in the mixed-shy group was similar to those of the negative-shy group and greater than in the positive-shy group, suggests that the negative expressions of shyness always denote a lack of regulation of shyness. These findings should be, however, considered with caution because the presence or absence but not the frequency of shy expressions was used as criterion.

In conclusion, children’s facial expressions can be indicators of their regulation of shyness, and of their level of social anxiety. It is likely that children have an innate predisposition to express their shyness in a positive or in a negative way. For instance, positive expressions of shyness have already been observed at the age of 3–4 months (Colonnesi et al. 2013; Reddy 2000). However, the expression of positive shyness may also be shaped by socialization since children are able to learn new social competences in their social development, during interactions with their parents, peers or other significant persons. Hence, they may also be able to learn positive shy expression, when they experience that positive expressions have positive social outcomes. We might therefor conclude that although positive and negative shyness start as unintentional expressions, children can learn to improve their approach to adaptively cope with their avoidance motivation (Li et al. 2016).

### Interplay of Theory of Mind and Expressions of Shyness as Predictor of Social Anxiety

As expected, children’s proclivity to express shyness in a positive way reduced the association between low ToM and social anxiety. Similarly, Banerjee and Henderson (2001) found that a negative relation between ToM and social anxiety level was present only in children with high levels of shy negative affect. These findings can also be interpreted in the opposite way with ToM acting as a moderator on the relation between shyness and anxiety. That is, children’s low level of positive expressions was found to be associated to a greater level of social anxiety, only when the level of ToM was low or medium but not when the level of ToM was high. In conclusion, the highest level of social anxiety was found in children who had a deficit in ToM and used few expressions of positive shyness whereas social anxiety was reduced when either ToM or positive shy expressions were high.

In line with previous finding among the relation between ToM and shyness (e.g., Suway et al. 2012) and shyness and social anxiety (e.g., Hirshfeld-Becker et al. 2007), children’s use of negative expressions of shyness were found to be related to a low level of ToM and to social anxiety without moderating this relation. Negative shy expressions represent social avoidance which could be the cause of a lower understanding of social interaction because avoiding social situations leads to less social experience. Similarly, a lower social understanding can lead to more negative social experiences and incapacity to maintain positive attitude in social situations (Findlay et al. 2009; Fordham and Stevenson-Hinde 1999). It should be noted that just as positive expressions of shyness, also the expressions of negative shyness can be a way to regulate arousal, an attempt to appease, and to regulate stressful social situations. However, the use of negative expressions of shyness seems to be only a short term solution. In the long run, avoiding social situations might enhance feelings of incompetence, and worries or fear for possible similar situations in the futures, favoring the onset of social anxiety (Findlay et al. 2009).

In conclusion, at the age of 4.5 the interplay between ToM and expressions of shyness can be used to detect children’s level of social anxiety. These findings should be, however, interpreted while keeping in mind the circularity of these relations. Hence, the level of social anxiety can be considered both as a result as well as a determinant of a low social understanding and low emotional regulation of self-conscious emotions.

### Limitations and Future Directions

The present study has some limitations that should be considered when interpreting the results. First, children’s expressions of shyness were observed only in one context, singing a song on stage, as the self-watching task did not elicit a sufficient number of expressions of shyness to be observed. To gain a wider insight into the role of expressions of shyness on children’s socio-emotional development, this behavior should be explored, next to social performance as we did, in social interactions with peers, as a core feature of social anxiety disorder. Second, by using a cross-sectional design we did not provide a developmental perspective of the associations between ToM, expressions of shyness and social anxiety. The associations should be further explored in the transition from childhood to adolescence, since the onset of social anxiety disorder often occurs in adolescence (Wittchen and Fehm 2001). Third, advanced ToM was assessed only with tasks tapping the understanding of belief and false-belief; no advanced understanding of emotions and desires were assessed in the present study.

The findings of the present study also offer important input for future research. The relations among ToM, expressions of shyness and social anxiety should be further explored taking into consideration children’s biological disposition (e.g., temperament) and environmental factors like significant social interactions (Carpendale and Lewis 2004). For instance, parental mentalization propensity towards the child, in terms of mind-mindedness (Meins et al. 2013), or reflective functioning (Sharp and Fonagy 2008), are found to be significant predictors of children’s secure attachment and ToM development (Taumoepeau and Ruffman 2006). Moreover, attachment has been found to play a significant role in children’s ToM development (Fonagy and Bateman 2006). Secure attachment seems, therefore, an important prerequisite for a stable and consistent representation of the self and of the others, which are crucial for a good self-organization and emotion regulation. Moreover, secure attachment has also been found to be negatively related to children’s development of social anxiety (meta-analysis of Colonnesi et al. 2011; *r* = 0.32). Parental mentalization towards the child, as well as parent-child attachment should therefore be explored in the relation between ToM, shyness, and social anxiety development. Other environmental factors that should be included in future investigations are parental rearing and parental psychopathology (Bögels et al. 2001). Also the question whether the ability to regulate shyness can be stimulated through the instruction of parents, teachers, and/or cognitive-behavioral or social skills interventions with children themselves, and what the best age is for such interventions, are questions for future research. A distinction between positive and negative expressions of shyness should be included in future instruments and procedures to detect shyness in order to distinguish between adaptive vs. non adaptive shy behavior.

## Conclusions

To conclude, a deficit in the development of social understanding and the onset of the first social-anxiety symptoms seem to be connected already in early childhood. Our findings also confirm the importance for children to develop adaptive coping strategies (i.e., expressing positive shyness rather than negative shyness) in order to cope with social anxiety, and to attenuate the effect of a lower social understanding. These results demonstrate an important relation between ToM development and social-emotional strategies, and how their interplay may prevent the onset of social anxiety symptoms.
